# Structural equation modeling approach to explore the influence of childhood maltreatment in adults

**DOI:** 10.1371/journal.pone.0239820

**Published:** 2020-10-01

**Authors:** Kuniyoshi Toyoshima, Takeshi Inoue, Jiro Masuya, Yota Fujimura, Shinji Higashi, Hajime Tanabe, Ichiro Kusumi

**Affiliations:** 1 Department of Psychiatry, Hokkaido University Graduate School of Medicine, Kita, Nishi, Sapporo, Japan; 2 Department of Psychiatry, Tokyo Medical University, Shinjuku-ku, Tokyo, Japan; 3 Department of Psychiatry, Tokyo Medical University, Hachioji Medical Center, Tokyo, Japan; 4 Department of Psychiatry, Ibaraki Medical Center, Tokyo Medical University, Ami-machi, Inashiki-gun, Ibaraki, Japan; 5 Department of Clinical Human Sciences, Graduate School of Humanities and Social Sciences, Shizuoka University, Ohya, Suruga-ku, Shizuoka, Japan; Chiba Daigaku, JAPAN

## Abstract

**Background:**

Childhood maltreatment affects social functioning in the general adult population. However, how child abuse affects functional disability in adulthood remains unknown. Thus, we investigated the correlation between child abuse, depressive symptoms, cognitive complaints, and functional disability in adult community volunteers.

**Methods:**

Participants (N = 556) completed the Child Abuse and Trauma Scale, Patient Health Questionnaire-9, Cognitive Complaints in Bipolar Disorder Rating Assessment, and Sheehan Disability Scale. Multiple regression analyses and structural equation modeling were performed to evaluate scale correlations.

**Results:**

Structural equation modeling showed that the direct effect of childhood maltreatment on depressive symptoms, the indirect effect of childhood maltreatment on cognitive function via depressive symptoms, and the indirect effects of childhood maltreatment on functional disability via depressive symptoms and via cognitive function were all significant. The direct effects of childhood maltreatment on cognitive function and functional disability were not significant. There was no significant association between variables.

**Limitations:**

Cross-sectional designs cannot identify causal relationships between parameters. Participants were adult volunteers from the community; therefore, results may not be generalizable to individuals with psychiatric disorders. Sociodemographic variability was a limitation because we used self-reported childhood maltreatment.

**Conclusions:**

Childhood maltreatment indirectly affects functional disability via depressive symptoms and via cognitive function through depressive symptoms. We suggest that depressive symptoms and cognitive function play crucial roles in the influence of childhood maltreatment on functional disability in adult community volunteers.

## Introduction

Childhood maltreatment affects the mental state of adults in the general population [1]. Previous research suggests that childhood maltreatment experiences increase the salience of stressful life events in adulthood [2]. Additionally, individuals who were first exposed to childhood maltreatment or interpersonal violence during middle childhood had more emotional dysregulation [3]. The correlation between childhood maltreatment and personality is complex [4], and childhood trauma exposure affects clinical psychopathology [5]. Different types of childhood maltreatment are interrelated and are associated with differing severity of psychological distress in adulthood. Childhood maltreatment has various effects on mental function during adulthood [6].

Exposure to trauma at different ages differentially impacts depressive symptoms during adulthood [7]. Childhood maltreatment impacts biological systems [8]. For example, previous research suggested that the short alleles of the serotonin transporter length polymorphism affect the correlation between childhood maltreatment and depression in adulthood [9]. Experience of the traumatic events in childhood doubles the risk of late-life depression and increases the risk of recurrent depressive episodes [10]. Furthermore, the influence of childhood neglect on the course of depression is considered to be independent of sociodemographic and clinical variables [11]. Recent research has suggested that an insecure attachment and parental maltreatment increased the risk of depression [12]. Therefore, childhood maltreatment has various effects on depressive symptoms in adulthood.

In some cases, childhood maltreatment influences depression through other mediators. Childhood maltreatment indirectly worsens depressive symptoms via affective temperaments or neuroticism [13, 14]. Moreover, childhood maltreatment not only directly affects mental disorders but also indirectly affects them through vulnerability characteristics [15]. The influence of childhood maltreatment on depression is mediated by several factors. Interpersonal sensitivity and trait anxiety mediate the influence of childhood maltreatment on depression in adulthood [16, 17]. Various factors influence the relationship between childhood maltreatment and adulthood depressive symptoms.

Regarding the correlation among childhood maltreatment, socioeconomic status, and adult memory, early stressful events affect cognitive processes [18]. However, the influence of childhood maltreatment on neurocognitive development is not fully elucidated [19]. Recent research has suggested that the experience of childhood violence victimization does not affect later cognition [20], whereas nonsexual maltreatment in childhood is associated with attention problems [21]. Cognitive function is correlated with neglect but not sexual or nonsexual abuse [22]. Furthermore, childhood neglect correlates with cognitive impairment in adulthood, independently of mental health [23]. Emotional neglect during childhood is particularly detrimental to memory in adulthood [24]. Thus, childhood maltreatment, especially neglect, plays an important role in cognitive impairment during adulthood.

Childhood maltreatment correlates with a poorer quality of life [25]. Adverse childhood experiences also correlate with poor general health and quality of life in adulthood [26, 27]. Childhood maltreatment also affects social functioning in adulthood [28]; moreover, it poorly affects the socioeconomic status in adulthood [22]. Childhood maltreatment is considered to determine the health-related quality of midlife in women [29] and correlates with intimate partner violence victimization in adulthood [30]. Regarding its mediator effects, childhood maltreatment indirectly worsens wellbeing via affective temperaments [31]. Furthermore, childhood maltreatment mediates the long-term consequences of household dysfunction in the general population [32]. Therefore, childhood maltreatment affects various aspects of social function in adulthood.

Depressive symptoms affect social function in the general adult population, and they may do so directly or indirectly via cognitive dysfunction [33]. Thus, cognitive function mediates the effect of depressive symptoms on social function. Recent research has suggested that cognitive complaints and depression severity affect social function, although objective cognitive impairments do not affect social function in major depressive disorder [34]. A relationship between cognitive complaints and social function has been reported in euthymic bipolar disorder [35]. There is an important correlation between social function and cognitive function, especially subjective cognitive function. Because childhood maltreatment influences depressive symptoms, cognitive function, and functional disability, childhood maltreatment may influence the mediator effect between these three variables [33] in addition to the moderator effect. However, to our knowledge, there is no evidence of this relationship.

We previously reported the relationship between depressive symptoms, congitive function, and social function in Japanese adults [33]. In the present study, we hypothesized that childhood maltreatment affects depressive symptoms, cognitive function, and functional disability and that depressive symptoms and cognitive function mediate the effect of child maltreatment on functional disability. According to this hypothesis and previous research, we performed a structural equation modeling to investigate whether depressive symptoms and cognitive complaints mediate the influence of childhood abuse on functional disability in adults from the community who volunteered for this study.

## Materials and methods

### Participants

All participants were recruited between April 2017 and 2018 at Tokyo Medical University in Japan. This research was part of a larger study on mental health in Japanese adults, in which several questionnaires were used a part of an investigation [33, 36]. This study was approved by the Local Ethics Committee of Tokyo Medical University (Ethics Approval Number: SH3502). After receiving a complete explanation of the research, written informed consent was provided by all participants (N = 597). Of this total, 556 participants completed all assessments, including demographic characteristics and evaluation scales.

### Assessments

Participants completed all assessments, including the Child Abuse and Trauma Scale (CATS) [37], Patient Health Questionnaire-9 (PHQ-9) [38], Cognitive Complaints in Bipolar Disorder Rating Assessment (COBRA) [39], and Sheehan Disability Scale (SDS) [40].

#### Childhood maltreatment measure

CATS is a 38-item self-assessment scale that assesses childhood maltreatment. It consists of a five-point assessment (0 = never, 1 = rarely, 2 = sometimes, 3 = very often, and 4 = always) [37]. For the present study, the Japanese version translated by Tanabe et al. [41] was used, and a total score of 38 items was calculated. The internal consistency of the overall CATS, as reflected in Cronbach’s alpha, was 0.90. The total score was calculated by adding each score rated on a five-point Likert scale (0 = never, 1 = rarely, 2 = sometimes, 3 = very often, and 4 = always). There were no missing data. It contains questions related to the individual’s childhood or adolescent experiences of sexual mistreatment, physical mistreatment and punishment, psychological mistreatment, physical or emotional neglect, and negative home environment (e.g., parental substance abuse or fighting). For example, one item asks, “Did your parents ridicule you”? [37]. The CATS scores correlated significantly (r = 0.44; p < 0.001) with scores on Bernstein and Putnam’s Dissociative Experiences Scale. The Gutman split-half reliability of CATS was 0.86 [37].

#### Depressive symptoms measure

PHQ-9 is a self-assessment scale that evaluates the depression severity [38]. A Japanese version was previously developed and exhibited good validity [42]. Scores were summed. This study used a summary score for evaluating the severity of depressive symptoms. In particular, we calculated the number of times (0–27 points) the patients experienced nine depressive symptoms in the previous two weeks using a four-point Likert scale for each item (0 = not at all, 1 = several days, 2 = more than half the days, and 3 = nearly every day). For the Japanese version of PHQ-9, the sensitivity (90.5%) and specificity (76.6%) were confirmed using the optimal cut-off points ≥10 for depression [43].

#### Subjective cognitive assessment

COBRA is a 16-item self-assessment scale that is used to evaluate neurocognitive functioning [39]. Items are assessed using a four-point scale (0 = never, 1 = sometimes, 2 = often, and 3 = always), and all items relate to daily life. A total score is calculated by summing all responses, with a maximum score of 48. Higher scores indicate higher levels of cognitive complaints. The validity and reliability of the Japanese version have been established [44]. COBRA is recommended for screening neurocognitive dysfunction of remitted bipolar patients [45]. Moreover, it can evaluate cognitive complaints in the general adult population [33]. In remitted bipolar disorder, a cut-off value > 14 of the COBRA total score is used to indicate moderate to severe self-reported difficulties [45]. These recommendations are for bipolar patients and not nonclinical individuals; however, the cut-off value is also helpful when using the COBRA in the general adult population [33]. The Japanese version of COBRA had very high internal consistency (Cronbach’s alpha = 0.887) for the total score [44].

#### Functional disability scale

SDS evaluates global functional disability and includes three items on disability that affect working, social functioning, and family living [40]. Participants were assessed on a 10-point visual analog scale for each item. The highest total value is 30, with the lowest value showing the least illness disruption [46].

### Statistical analyses

There were no missing data, and no multicollinearity was present in multiple regression analysis. Multiple regression analyses (by the forced-entry method) were performed using the SDS total score as the dependent variable, with COBRA, PHQ-9, CATS, and clinical parameters as independent variables; with COBRA total score as the dependent variable and PHQ-9, CATS, and clinical parameters as independent variables; and with PHQ-9 summary score as the dependent variable and CATS and clinical parameters as independent variables. Before assessing the interaction, centering was performed on the mean scores. A covariance structure analysis was subsequently performed to investigate the relationship between the parameters using maximum likelihood robust estimation. The model was evaluated according to the multiple fit criteria of the Tucker–Lewis index (TLI), the comparative fit index (CFI), and the root-mean-square error of approximation (RMSEA). We used the following criteria: TLI above 0.97, a CFI above 0.97, and an RMSEA below 0.05 show a good fit [47]. Mediation analysis was performed using the robust maximum likelihood estimation method. All statistical analyses, except for structural equation modeling, were performed using STATA/MP 16 (Stata Corp, LLC, College Station, TX), and structural equation modeling was performed using Mplus version 8.4 (Muthén & Muthén, Los Angeles, CA, USA); *p* < 0.05 was considered statistically significant.

## Results

A total of 556 nonclinical adult participants were included in this study (Table 1).

**Table 1:** Clinical and sociodemographic data (N = 556).

	Mean (SD) or n (%)
Age, mean (SD)	41.2 (11.9)
Sex [male], n (%)	243 (43.7)
Married, n (%)	369 (66.4)
Years of education, mean (SD)	14.7 (1.8)
Current employment, n (%)	547 (98.4)
Psychiatric history, n (%)	58 (10.4)
Current psychiatric treatment n (%)	22 (4.0)
Drinking alcohol, n (%)	357 (64.2)
Smoking, n (%)	105 (18.9)
CATS total score, mean (SD)	27.2 (20.2)
PHQ-9 summary score, mean (SD)	4.1 (4.2)
SDS work score, mean (SD)	2.0 (2.5)
SDS social score, mean (SD)	1.7 (2.5)
SDS family score, mean (SD)	1.5 (2.4)
SDS total score, mean (SD)	5.3 (6.7)
COBRA total score, mean (SD)	8.3 (6.6)
COBRA total score >14, n (%)	96 (17.3)

Abbreviations: CATS, Child Abuse and Trauma Scale; COBRA, Cognitive Complaints in Bipolar Disorder Rating Assessment; PHQ-9, Patient Health Questionnaire-9; SDS, Sheehan Disability Scale.

### Relationship between childhood maltreatment, depressive symptoms, cognitive function, and functional disability

Spearman’s rank correlation analysis showed that there were significant relationships between the CATS total score and the PHQ summary score, COBRA total score, and SDS total score (Table 2). The associations among the PHQ-9 summary score, COBRA total score, and SDS total score were statistically significant. The COBRA total score significantly correlated with SDS total score (Table 2). The correlation between childhood maltreatment, depressive symptoms, cognitive function, and functional disability was statistically significant.

**Table 2:** Spearman’s rank correlations (*rho*) between CATS, PHQ-9, COBRA, and SDS (N = 556).

	CATS	PHQ-9	COBRA	SDS total
CATS	-			
PHQ-9	0.34**	**-**		
COBRA	0.29**	0.41**	**-**	
SDS total	0.34**	0.56**	0.39**	-

Abbreviations: CATS, Child Abuse and Trauma Scale; COBRA, Cognitive Complaints in Bipolar Disorder Rating Assessment; PHQ-9, Patient Health Questionnaire-9; SDS, Sheehan Disability Scale, *p* < 0.01** (two-sided).

### Relationship between demographic characteristics, child abuse, depressive symptoms, cognitive function, and functional disability

Multiple regression analysis—with the SDS total score as the dependent variables and demographic data, CATS total score, PHQ-9 summary score, and COBRA total score as the independent variables—was performed. The CATS score, PHQ-9 score, and COBRA total score significantly predicted the SDS total score (Table 3).

**Table 3:** Multiple regression analysis (*β*) of SDS, COBRA, PHQ-9, and CATS (N = 556).

	SDS total	COBRA	PHQ-9	CATS
Independent variables	F (12, 543) = 26.53, *p *< 0.0001	F (11, 544) = 11.58, *p *< 0.0001	F (10, 545) = 12.59, *p *< 0.0001	F (9, 546) = 4.50, *p *< 0.0001
Age	0.03	0.08	−0.03	−0.02
Sex: 1 (Male); 2 (Female)	0.01	0.04	0.06	0.06
Married: 1 (No); 2 (Yes)	−0.04	−0.05	−0.16**	−0.03
Years of education	0.00	0.03	−0.01	−0.19**
Currently employed: 1 (No); 2 (Yes)	0.04	−0.07	0.02	−0.02
Psychiatric history: 1 (No); 2 (Yes)	0.05	0.03	0.18**	0.07
Current psychiatric treatment: 1 (No); 2 (Yes)	0.04	0.07	0.06	0.08
Drinking alcohol: 1 (No); 2 (Yes)	−0.01	0.05	−0.05	−0.03
Smoking: 1 (No); 2 (Yes)	−0.03	−0.03	−0.01	0.03
CATS	0.10**	0.04	0.27**	-
PHQ-9	0.47**	0.37**	-	-
COBRA	0.12**	-	-	-
**Adjusted *R***^***2***^	0.36	0.17	0.17	0.05

Abbreviations: CATS, Child Abuse and Trauma Scale; COBRA, Cognitive Complaints in Bipolar Disorder Rating Assessment; PHQ-9, Patient Health Questionnaire-9; SDS, Sheehan Disability Scale, *p* < 0.05*, *p* < 0.01** (two-sided).

Multiple regression analysis—with the COBRA total score as the dependent variable, and demographic data, CATS total score, and PHQ-9 summary score as the independent variables—was performed. The PHQ-9 score significantly predicted the COBRA total score (Table 3).

Multiple regression analysis—with the PHQ-9 summary score as the dependent variable and demographic data and CATS total score as the independent variables—was performed. Marital status, psychiatric history, and CATS score significantly predicted the PHQ-9 summary score (Table 3).

Multiple regression analysis by forced injection method—with the CATS total score as the dependent variable and demographic data as the independent variables—was performed. Years of education significantly predicted the CATS total score (Table 3).

Hierarchical multiple regression analysis was performed to evaluate the interaction between child maltreatment, depressive symptoms, and cognitive function. No significant interaction was observed between the parameters (Table 4).

**Table 4:** Results of the hierarchical multiple regression analysis of SDS and COBRA (N = 556).

	SDS total score			COBRA		
Variable	*β*	*p*	B	*β*	*p*	B
*Step 1*	F (3, 552) = 103.160, *p* = 0.000			F (2, 553) = 56.016, *p* = 0.000		
CATS	0.100	0.006	0.033	0.045	0.267	0.015
PHQ-9	0.498	0.000	0.791	0.394	0.000	0.618
COBRA	0.124	0.001	0.126			
Adjusted *R*^*2*^		0.356			0.165	
*Step 2*	F (6, 549) = 52.343, *p* = 0.000			F (3, 552) = 38.725, *p* = 0.000		
CATS	0.117	0.003	0.038	0.066	0.117	0.022
PHQ-9	0.489	0.000	0.777	0.414	0.000	0.650
COBRA	0.115	0.003	0.117			
CATS × PHQ-9	−0.059	0.160	−0.004	−0.081	0.058	−0.005
CATS × COBRA	−0.026	0.537	−0.001			
PHQ-9 × COBRA	0.065	0.142	0.012			
⊿*R*^*2*^		0.005 (*p* = 0.261)			0.005 (*p* = 0.058)	
Adjusted *R*^*2*^		0.357			0.169	

Abbreviations: CATS, Child Abuse and Trauma Scale; COBRA, Cognitive Complaints in Bipolar Disorder Rating Assessment; PHQ-9; Patient Health Questionnaire-9; SDS, Sheehan Disability Scale.

In summary, child maltreatment, depressive symptoms, and cognitive dysfunction significantly predicted functional disability. Cognitive function was significantly impacted by depressive symptoms, and depressive symptoms were significantly impacted by marital status, psychiatric history, and childhood maltreatment. Years of education significantly predicted childhood maltreatment.

### Structural equation modeling

**Fig 1:**
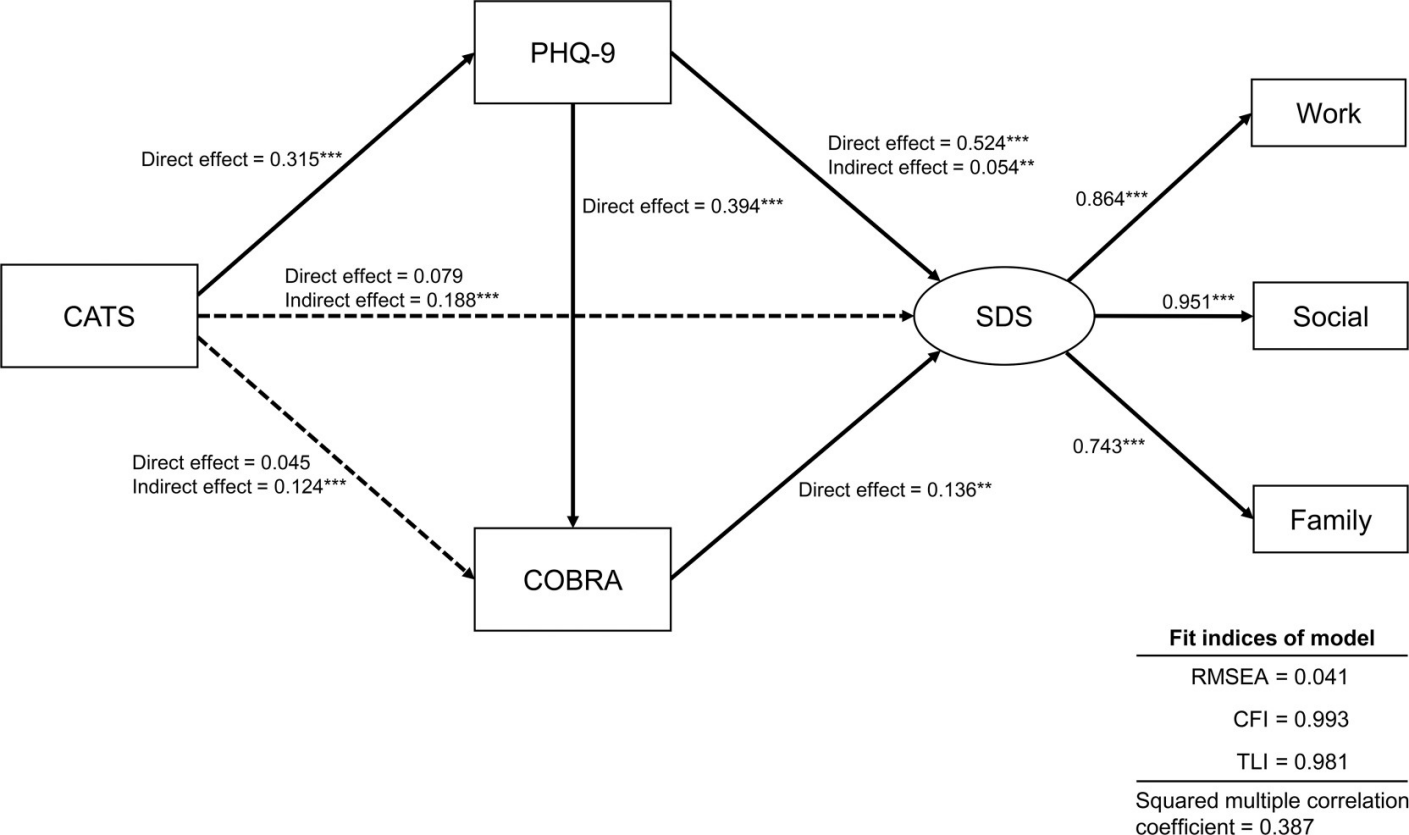
Structural equation model. *Abbreviations*: CATS = Child Abuse and Trauma Scale, COBRA = Cognitive Complaints in Bipolar Disorder Rating Assessment, PHQ-9 = Patient Health Questionnaire-9, SDS = Sheehan Disability Scale, Work = SDS work, Social = SDS social, Family = SDS family, RMSEA = root-mean-square error of approximation; CFI = comparative fit index; TLI = Tucker–Lewis index. “SDS” was the latent variable, comprising three observed variables: SDS work, social, and family. The numbers next to the arrows show the standardized path coefficients (minimum −1, maximum 1). The rectangles represent the observed variables, and the oval shows the latent variable. The solid lines indicate statistically significant paths, and the broken line indicates the non-significant path. *p < 0.05, **p < 0.01, ***p < 0.001 (two-sided).

To evaluate the correlations between childhood maltreatment, depressive symptoms, cognitive function, and functional disability, we performed a structural equation modeling on the basis of the results of the multiple regression analyses. Fig 1 shows this equation, as well as the results of the standardized path coefficients. Fit indices revealed a good fit (RMSEA = 0.041, CFI = 0.993, and TLI = 0.981). In this model, “SDS” was the latent variable, comprising three observed variables: SDS work, social, and family. The model explained 38.7% of the variability of cognitive complaints (squared multiple correlation coefficient = 0.387).

Regarding direct effects, cognitive function (0.136, *p* < 0.01) and depressive symptoms (0.524, *p* < 0.001) had significant effects on “Disability.” (Table 5). Depressive symptoms (0.394, *p* < 0.001) had a significant direct effect on cognitive function, whereas childhood maltreatment did not (0.045, *p* > 0.05). Childhood maltreatment had a significant direct effect on depressive symptoms (0.315, *p* < 0.001). Depressive symptoms (0.054, *p* < 0.01) and childhood maltreatment (0.188, *p* < 0.001) had significant indirect effects on “Disability.” According to the mediation analysis, the indirect effects of childhood maltreatment via depressive symptoms (0.165, *p* < 0.001) and via depressive symptoms and cognitive function (0.017, *p* < 0.01) on “Disability” were statistically significant, whereas the indirect effects of childhood maltreatment via cognitive function (0.006, *p* > 0.05) on “Disability” was not statistically significant. Childhood maltreatment had significant indirect effects on cognitive function via depressive symptoms (0.124, *p* < 0.001). Table 5 shows the significant indirect path from childhood maltreatment to “Disability”, namely, “childhood maltreatment–depressive symptoms–disability” and “childhood maltreatment–depressive symptoms–cognitive function–disability.”

**Table 5:** Standardized path coefficients between each variable (N = 556).

	Direct effect to		
From	PHQ-9	COBRA	SDS
CATS	0.315***	0.045	0.079
PHQ-9		0.394***	0.524***
COBRA			0.136**
	Indirect effect to		
	PHQ-9	COBRA	SDS
CATS		0.124***(via PHQ-9)	0.165***(via PHQ-9)
			0.017**(via PHQ-9 and COBRA)
			0.006 (via COBRA)
	Total indirect effect	0.124***	0.188***
PHQ-9			0.054** (via COBRA)
	Total effect to		
	PHQ-9	COBRA	SDS
CATS	0.315***	0.169**	0.267***
PHQ-9		0.394***	0.578***
COBRA			0.136**

Abbreviations: CATS, Child Abuse and Trauma Scale; COBRA, Cognitive Complaints in Bipolar Disorder Rating Assessment; PHQ-9; Patient Health Questionnaire-9; SDS, Sheehan Disability Scale, “SDS” was the latent variable, comprising three observed variables: SDS work, social, and family. *p* < 0.05*, *p* < 0.01**, *p* < 0.001*** (two-sided).

In summary, childhood maltreatment affected depressive symptoms directly, and depressive symptoms affected cognitive function directly. Depressive symptoms affected functional disability directly and indirectly via cognitive function. Childhood maltreatment affected cognitive function indirectly via depressive symptoms and affected functional disability indirectly via depressive symptoms and via depressive symptoms and cognitive complaints. In summary, childhood maltreatment affected depressive symptoms, and cognitive complaints mediated the effect of depressive symptoms on functional disability.

## Discussion

The present study used structural equation modeling to show the relationship between childhood maltreatment, depressive symptoms, cognitive impairment, and functional disability. Our results demonstrate that childhood maltreatment indirectly affects functional disability via depression and cognitive function. Interestingly, the direct effects of childhood maltreatment on cognitive function and functional disability were not statistically significant, while the direct effect of childhood maltreatment on depressive symptoms was statistically significant. In the structural equation model, childhood maltreatment affected cognitive function and functional disability only via depressive symptoms. Therefore, we suggest that depressive symptoms are important in the correlation between childhood maltreatment and cognitive function or functional disability in Japanese adults.

This study demonstrates that childhood maltreatment affects depressive symptoms directly. This finding is consistent with the results of previous research [17]. According to the previous study, trait anxiety mediates the effects of childhood maltreatment on depression in the general adult population [17]. We recently investigated the relationships between affective temperaments, subjective cognitive function, and social function, and showed that subjective cognitive function mediates the influence of affective temperaments on social function [48]. In this study, assessments on affective temperaments or anxiety have not been performed, which is a limitation of this study. Regarding sociodemographic characteristics, multiple regression analysis shows that childhood maltreatment, marital status, and psychiatric history predict depressive symptoms (Table 3). Depressive symptoms are generally affected by various kinds of sociodemographic factors. In this study, most individuals were employed during the assessment, which limits our conclusions to people who were not employed, because employment status affects social functions

According to previous research, trauma exposure at different ages differentially impacts depressive symptoms in adulthood [7]. In this study, the age of childhood maltreatment was not controlled. This is also a limitation of this research. The results of the multiple regression analysis show that only years of education predict child maltreatment (Table 3). Years of education correlate with intelligence quotient (IQ); therefore, we speculate that childhood maltreatment correlates with IQ. Regarding the relationship between childhood violence victimization and IQ, previous research suggested that a low IQ in victimized subjects was largely contributed by cognitive dysfunction which predated childhood maltreatment [20]. In this study, years of education did not significantly predict subjective cognitive impairment, which means acquired deterioration (Table 3). Years of education was also strongly associated with socioeconomic status, and research has shown that child maltreatment occurs more in people with low socioeconomic status [49]. Therefore, individuals with a low IQ may tend to have an experience of childhood maltreatment.

Previous research suggested that the patterns of childhood maltreatment correlate with the severity of mental illness in adulthood [50]. In this study, the patterns of childhood maltreatment were not considered; that is, we used the data of the CATS total score. Therefore, we cannot discuss different types of childhood maltreatment. This is another limitation of this study. Recent research suggested that insecure attachment and parental maltreatment increased the risk of depression [12]. Future studies should investigate the complex relationships between types of childhood maltreatment and adulthood depressive symptoms.

This research shows that childhood maltreatment indirectly affects cognitive function via depressive symptoms. Recent studies suggested that childhood neglect affects cognitive dysfunction in adulthood [22–24], whereas the correlation among childhood violence victimization and later cognitive function is considered to be non-causal [20]. In this study, childhood maltreatment includes both neglect and abuse, which may be affected by the non-significance of the direct effect on cognitive function in the structural equation model. Although this is also a limitation of this study, we found an indirect effect of child maltreatment on cognitive complaints through depressive symptoms in our model. Our results demonstrate the indirect effect of child maltreatment on cognitive complaints in adult community volunteers. Previous research suggested that childhood neglect particularly affects memory function in adulthood [24]. Future studies should study the correlation among childhood maltreatment, depressive symptoms, and memory function in Japanese adults.

This study demonstrates that childhood maltreatment indirectly affects functional disability. In the structural equation model, cognitive function mediates the effect of depressive symptoms on functional disability (Fig 1). The mediator effect of cognitive function between depressive symptoms and life quality has been reported in our previous research [33]. Previous studies suggested that childhood maltreatment affects socioeconomic outcomes and household dysfunction in adulthood [22, 32]. In this research, according to the outcomes of the multiple regression analysis (Table 3), the effects of functional disability were significantly predicted by childhood maltreatment. Thus, childhood maltreatment may correlate with adulthood functional disability.

Regarding the mediator effect on childhood maltreatment, affective temperaments mediate the effects of childhood maltreatment on depression, especially neglect [13]. Vulnerability characteristics mediate the effect of childhood maltreatment on incident mental disorders [15]. Interpersonal sensitivity mediates the effect of childhood maltreatment on depression in adulthood [16]. In addition, trait anxiety mediates the effect of child maltreatment on depression in adulthood [17]. In this study, the moderator effects of childhood maltreatment on cognitive function or functional disability have not been shown (Table 4). Regarding the relationship between the moderator effect and childhood maltreatment, the moderator roles of affective temperaments, child maltreatment, and adulthood life events on depression have been reported [51]. Future studies should investigate the moderator effects of childhood maltreatment and depressive symptoms on cognitive complaints in a larger sample.

### Limitations

Self-report measures were used in this study, which are subject to bias. In addition, a retrospective measure of child maltreatment was used in this study, whereas research has shown that the correlation between retrospective and prospective measures of child maltreatment is low. Retrospective self-reports of childhood maltreatment may need to consider the current mood state to account for potential memory biases [52]. Moreover, mediation analyses was performed whereas all measures were concurrently measured. This study cannot identify causal relations among the parameters because this research followed a cross-sectional design. The participants were adult volunteers from the community. This limits the generalizability of the results to other communities. In addition, the participants were adults from the community recruited using convenience sampling in Japan; therefore, our conclusion may not be applicable to other communities, children, or adolescents. Other limitations include the heterogeneous characteristics of the volunteers, including a range of healthy and unhealthy individuals from diverse social and educational backgrounds. Moreover, we could not control for the effect of medication and mood symptoms on measures at the time of the assessment. Furthermore, we used self-assessments; therefore, childhood maltreatment, depressive symptoms, cognitive function, and social function should also be evaluated using objective assessments in future studies. Most participants (98.4%) were employed at the time of the study; thus, it was not an economically deprived group, and therefore, the sample was skewed to include more functional adults who could hold down a job. This may also limit the variability of cognitive functioning within the sample and its interaction with the other variables. Finally, sociodemographic variability is a limitation because we used self-rating assessment on childhood maltreatment in the community adults [53].

## Conclusions

Our results reveal that childhood maltreatment affects depressive symptoms, and cognitive function mediates the effect of depressive symptoms on functional disability in adult volunteers from the community. Childhood maltreatment affects functional disability indirectly via depressive symptoms and via cognitive function through depressive symptoms. We conclude that depressive symptoms and cognitive function play important roles in the effect of childhood maltreatment on functional disability in adult community volunteers.

## Supporting information

S1 Checklist STROBE statement—checklist of items that should be included in reports of observational studies. (DOCX)
